# Of carrots and sticks: the effect of workfare announcements on the job search behaviour and reservation wage of welfare recipients

**DOI:** 10.1186/s12651-018-0245-9

**Published:** 2018-10-22

**Authors:** Katrin Hohmeyer, Joachim Wolff

**Affiliations:** Institute for Employment Research, Regensburger Straße 104, 90478 Nuremberg, Germany

**Keywords:** Activation, Employment, Intention-to-treat effect, Job search, Threat effect, Attraction effect, Propensity score matching, Welfare receipt, Workfare, I38, J64, J68

## Abstract

The German workfare scheme ‘One-Euro-Jobs’, which provides additional jobs of public interest for welfare recipients, has a number of different goals. On the one hand, One-Euro-Jobs are intended to increase the participants’ employment prospects in the medium term. On the other hand, they can be used to test welfare recipients’ willingness to work. We use survey data from the Panel Study ‘Labour Market and Social Security’ and propensity score matching methods to study the intention-to-treat effect of receiving a One-Euro-Job announcement on job search behaviour, reservation wage and labour market performance of welfare recipients. We find that receiving a One-Euro-Job announcement increases job search activities significantly and decreases the reservation wage for women and individuals who have been employed within the last 4 years, but does not affect the short-term employment probability.

## Introduction

Unemployment benefits provide individuals with income support in the case of unemployment, but also reduce work incentives. Job search requirements and active labour market programmes (ALMPs) can increase work incentives because they can make unemployment benefit receipt less attractive. The German Hartz reforms in the early 2000s implemented a principle of rights and duty and implied a shift towards activation (Eichhorst et al. 2010). Benefit recipients are required to take up any job or participate in ALMPs. Moreover, more possibilities than before the reforms are available to assist unemployed individuals in taking up a job. Activation policies thus have enabling as well as demanding elements. This is particularly evident for One-Euro-Jobs, a large-scale workfare programme for welfare recipients. One-Euro-Jobs are temporary jobs, which have to be additional and of public interest. On the one hand, they aim at improving employment prospects of hard-to-place individuals, who ideally get used to regular work schedules again and improve their social integration during participation. On the other hand, One-Euro-Jobs can be used to test welfare recipients’ willingness to work.

Evaluation studies found lock-in effects of participating in a One-Euro-Job in the short term and moderate positive effects on the medium-term employment prospects for several groups of participants (e.g., Dengler 2015; Hohmeyer 2012; Hohmeyer and Wolff 2012). Studies on actual participation cannot capture the full effect that an expected future participation can have: Not all welfare recipients who are subject to an announcement of a One-Euro-Job participation will later participate. We will examine the effect of such an assignment and hence study an intention-to-treat effect that not only captures effects for compliers but also for non-compliers and dropouts (Gupta 2011).^1^ Specifically, we use propensity score matching to study the effect of receiving a One-Euro-Job announcement on the job search behaviour, the reservation wage and the short-term labour market performance of welfare recipients receiving such an announcement. The intention-to-treat effect will hence not only capture participation effects, but will also encompass “pure” announcement effects. The sign of these effects is theoretically ambiguous. These can be threat effects (Bjørn et al. 2005), e.g., if people would like to avoid a potential One-Euro-Job programme participation because they regard it as detrimental. They might for instance regard participation as an adverse signal to employers or detrimental for their employability. In turn, they could intensify their job search activities and lower reservation wages to circumvent or stop participation in an announced One-Euro-Job by taking up regular jobs or retreating from the labour market. Other welfare recipients might have a different view and expect that One-Euro-Job participation raises their employability. Moreover, they might derive some direct utility from participation [e.g., due to the psychosocial functions of work (Jahoda 1982)]. Correspondingly, Knabe et al. (2017) find that One-Euro-Job participants have a higher life satisfaction than unemployed people. In response, participants could reduce their search activities and increase reservation wages due to the pure announcement of a potential One-Euro-Job participation (also known as “attraction effect”) and due to participation itself. Overall, it is important not just to study effects of One-Euro-Job participation but the intention-to-treat effects for people who due to an announcement are expected to participate. A comprehensive assessment of programme effects must also include intention-to-treat effects.

This paper is the first to provide evidence on intention-to-treat effects of the large-scale German workfare programme. Our estimates of these effects are based on data surveying One-Euro-Job announcements and their timing directly within the Panel Study ‘Labour Market and Social Security’ (PASS). As our outcomes are measured at the interview after the announcements, a participation in One-Euro-Jobs might already have followed the announcement prior to that interview. We find that receiving a One-Euro-Job announcement that might or might not be followed by a participation leads to more intense job search activities and decreases the reservation wage for the treated, whereas we find no intention-to-treat effects on the short-term employment probability.

Section 2 turns to key features of the relevant institutional framework. Section 3 discusses the theoretical background of our study and related previous empirical evidence. Section 4 presents our data and the applied methods, while Sect. 5 presents the results of our analysis. Section 6 concludes.

## Institutional framework

In 2005, the last step of the Hartz reforms merged the former unemployment assistance and social assistance to form a new means-tested welfare benefit (Unemployment Benefit II, UB II) for needy individuals capable of working.^2^ One aim of the reform was activating a broad group of needy individuals with the goal of integrating them into the labour market (Eichhorst et al. 2010). Compared with other countries, being capable of working is defined very broadly as by being able to work for at least 3 h per day. Neediness is determined on household level (*Bedarfsgemeinschaft*). In contrast to the former system of unemployment assistance, all members of a needy household capable of working are in principle supposed to help reduce the household’s dependence on welfare benefits. The basic principles of the system are “*Fördern* (enabling)” and “*Fordern* (demanding)”, i.e. supporting the jobseekers on the one hand and demanding individual effort on the other hand. The introduction of a workfare programme called One-Euro-Jobs was important in this context. On the one hand, One-Euro-Jobs played a major role in the Hartz IV reform as a demanding element. Welfare recipients can be assigned to the programme to provide work in return for their benefit. One-Euro-Job participation hampers working in the shadow economy for welfare recipients. Thus, welfare recipients are provided with additional incentives to search for regular work that can easily yield a higher income than participation in One-Euro-Jobs. On the other hand, One-Euro-Jobs can represent an enabling element, if the welfare recipients with very low job finding prospects are assigned to the programme and improve their employability and social integration by this participation.

In the first years after their introduction, One-Euro-Jobs were one of the most widely used ALMPs in Germany. Between 2006 and 2009, more than 700,000 welfare recipients started the programme per year. Table 1 confirms the importance of One-Euro-Job participation in our observation period 2009–2013 also compared with other programmes. In 2009, the annual average stock of welfare recipients was roughly 4.8 million people and more than 720,000 welfare recipients entered a One-Euro-Job. The inflow into other ALMPs is considerable, but much lower than the inflow into One-Euro-Jobs. Only schemes for activation and integration that represent a heterogeneous programme consisting of short trainings and private placement services are characterised by a higher inflow than One-Euro-Jobs since 2010. The inflow into One-Euro-Jobs decreased in the following years to about 200,000 new participants in 2017 (Department for Statistics of the German Federal Employment Agency 2018).

**Table 1:** Inflow of unemployment benefit II recipients into main ALMPs and annual average stock of unemployment benefit II recipients (in 1000). Source: Statistics Department of the German Federal Employment Agency

Year	2009	2010	2011	2012	2013
Private placement services	106.5	–	–	–	–
Short training	256.7	–	–	–	–
Schemes for activation and integration^a^	550.8	960.9	781.2	750.6	775.4
Further vocational training	265.3	255.3	184.6	195.1	176.0
General employer wage subsidy	127.3	141.1	115.1	87.0	77.4
Start-up subsidy	19.8	16.7	11.2	7.9	5.9
One-Euro-Jobs	722.6	660.4	436.1	342.9	278.9
Different subsidies to employ people who are hard-to-place	125.9	88.8	64.1	29.8	16.9
Stock of unemployment benefit II recipients	4866.0	4837.8	4565.0	4402.9	4389.8

^a^This ALMP was introduced in 2009 to replace the private placement services and short training programme

One-Euro-Jobs are temporary jobs, which are supposed to be additional and of public interest and from April 2012 should not affect competition among companies. These requirements should prevent negative effects on regular employment and windfall gains of subsidized employment. Before and after 2012, One-Euro-Jobs consisted mostly of community service jobs. Moreover, the tasks of One-Euro-Jobbers should mainly be tasks that would not be carried out by regular employees, like organizing and participating in leisure activities for people in a home for the elderly. In our observation period 2009–2013, more than half and sometimes more than 60 per cent of the annual One-Euro-Job inflow were in the fields environment protection, rural conservation and infrastructure improvement (Data warehouse of the Department for Statistics of the German Federal Employment Agency). This includes tasks such as the maintenance of public parks or play grounds. With a share of 16–19 per cent of the annual inflow in 2009–2013, another important field of work was also health and child care/youth welfare. People performing One-Euro-Jobs continue to receive their welfare benefit plus one to two Euros per hour worked as an allowance for their additional expenses.

Participation in a One-Euro-Job can have various goals. One of them is to increase the medium-term employment prospects of participants. Given that the programme is designed to provide additional jobs of public interest, this goal is mainly pursued by providing participants, who often have been out of work for several years, with social contacts and a daily routine. This goal is related to improving the social integration of participating welfare recipients. Another goal of One-Euro-Jobs is to make welfare recipients reciprocate for receiving their benefit. Once assigned to the programme, participation is compulsory and benefits can be cut in the case of non-participation without good reason (Wolff and Moczall 2012). Overall, One-Euro-Jobs have an ambivalent character and include both “carrot” and “stick” elements.

Participation is subordinate to regular employment or participation in other ALMPs. Given this “last resort” character and the design of the programme, the primary target group of the programme comprises hard-to-place individuals who cannot find a job otherwise. The Federal Employment Agency (2004) lists the following groups as potential target groups for activation because they on average tend to have specific difficulties finding a job: migrants, women, disabled persons, long-term unemployed and young people under 25 and the older unemployed. However, given the potential use of One-Euro-Jobs as a work test, welfare recipients with good labour market prospects can be a target group of the programme as well.

There are no explicit rules that determine when during his or her spell of benefit receipt a welfare recipient should participate in a One-Euro-Job. Whether and when a welfare recipient is assigned to a One-Euro-Job largely lies at the discretion of the case worker. Evidence on the selectivity of One-Euro-Jobs concludes that especially in the first year after the introduction of One-Euro-Jobs when the programme was very widely used, most of the target groups of hard-to-place unemployed were not reached yet (see, e.g., Hohmeyer and Kopf 2009; Thomsen and Walter 2010). Further evidence looking at different direct job creation schemes indicates that One-Euro-Jobs may not focus on hard-to-place individuals among the welfare recipients but that they are used subordinately to other programmes (Hohmeyer and Wolff 2010). Different steps can lead to assignment to and participation in a One-Euro-Job (Hohmeyer and Kopf 2009). In a typical assignment procedure, the first step would be that the welfare recipient and the case worker talk about One-Euro-Job participation in general. In about two-thirds of the cases, the caseworker mentions One-Euro-Jobs as a topic first (Hohmeyer and Wolff 2015). Eventually, the caseworker suggests a concrete One-Euro-Job for participation. Often, a job interview takes place in the operating establishment. The final participation in the programme always works through a written assignment to a concrete One-Euro-Job. Non-participation in a reasonable One-Euro-Job that the welfare recipients has been assigned to can be sanctioned. About 58% actually participate in the One-Euro-Job the caseworker announced to them.^3^ Main reasons for non-participation are illness, disinterest and rejection by the establishment (Hohmeyer and Wolff 2015). Only in a minority of cases is taking up employment or leaving welfare receipt the reason for non-participation.

## Announcement and participation effects of One-Euro-Jobs: theoretical considerations and previous evidence

The intention-to-treat effect of receiving a One-Euro-Job announcement comprises effects of the treatment announcement as well as the participation itself. The framework of the job search model enables us to discuss both types of effects on the job search behaviour, reservation wages (the lowest wage that they will accept) and labour market outcomes of participants. In the basic job search model with endogenous search effort, unemployed individuals maximize their expected utility by choosing the reservation wage and the job search intensity (Cahuc and Zylberberg 2004). The reservation wage is determined by the gains (e.g., unemployment benefits) and costs associated with job search periods, the labour market state, the arrival rate of job offers and the real interest rate. In each short period, jobs disappear with a fixed rate. The real wage is the only relevant aspect of jobs offered. The job seekers do not know the exact wage each job pays but only the cumulative distribution of possible wages. The expected unemployment duration depends positively on the reservation wage and negatively on the arrival rate of job offers, which are themselves influenced by factors such as job search intensity or ALMP participation.

Participation in an ALMP itself can affect job search behaviour and labour market outcomes (Calmfors 1994). In the short term, lock-in effects can reduce participants’ job search efforts, who have less time or less motivation to search for a job during participation. In the medium term, ALMP participation can increase the arrival rate of job offers because it leads to an update of welfare recipients’ qualifications or because it signals potential employers the participant’s willingness to work. However, adverse effects also can occur: The programme itself could lead to stigmatisation of participants, if employers possibly do not regard the programme as equivalent to regular employment or other forms of qualification. Furthermore, participation can also increase the reservation wage and thus decrease the probability of taking up a job. Several empirical evaluation studies found lock-in effects of participating in a One-Euro-Job in the short term and moderate positive effects on the medium-term employment prospects for several groups of participants (i.e. who have not worked for several years or are above 50 years of age) (e.g., Dengler 2015; Hohmeyer 2012; Hohmeyer and Wolff 2012).

Not only actual participation can affect individual behaviour but also its announcement. To study announcement effects of ALMPs, van den Berg et al. (2009) integrate the perceived participation probability in an ALMP and the expected treatment effect into the job search model framework. The announcement effect depends on how unemployed people perceive programme participation: If the expected gain of the participation is positive, then a positive perceived participation probability leads to a decrease in job search and an increase in the reservation wage (attraction effect). If welfare recipients expect a loss by participation, then the search intensity increases and the reservation wage decreases, because welfare recipients intend to avoid participation (threat effect). We assume that a One-Euro-Job announcement increases the perceived participation probability. The announcement then leads to an attraction effect, if welfare recipients expect the One-Euro-Job participation to be beneficial. If recipients of the announcement expect the One-Euro-Job participation to harm their job finding rate and the arrival rate of job offers, a threat effect occurs.

Even before an announcement of participation, attraction or threat effects might influence the job search behaviour of welfare recipients ex ante, who know that an assignment to the programme is possible. Once a participation is announced to them, these effects should become stronger. Furthermore, initial threat or attraction effects could be amplified or weakened by actual participation: The participation itself might imply that the participants perceive it as less or more beneficial than before. In turn, empirical studies (as the ones on One-Euro-Jobs participation effects that we briefly discussed) do not measure a pure participation effect but also include the influence of attraction and threat effects.

With respect to empirical evidence, we find that despite an increasing evidence on ex ante effects of ALMPs generally, no evidence on effects of One-Euro-Job announcements in Germany exists so far. Closely related to our approach are studies that investigate the effect of receiving a concrete announcement of participation in an ALMP.^4^ Most of these studies are based on randomised experiments. One exception is a study by Crépon et al. (2018), who use administrative data on training notifications in Paris. They find training notifications to lead to a lower probability of leaving unemployment (attraction effect).

Using data from the ‘Worker Profiling and Reemployment Services’, Black et al. (2003) find a sharp increase in early exits from unemployment insurance benefit receipt, after benefit recipients had been informed about their programme participation. Similarly, using data from Denmark, Graversen and van Ours (2008) find that being assigned to a mandatory activation programme increases job finding rates of newly unemployed. Using data from three experiments in Sweden, Hägglund (2011) provides evidence on increased exit rates from unemployment insurance benefit receipt due to programme assignment in Jämtland, where a broad group of unemployment insurance recipients was targeted. In contrast, he does not find well-determined threat effects in Uppsala and Östergötland, where locally specific groups were targeted. To the best of our knowledge, only one study for Germany is based on experimental data. Büttner (2008) observes that an announcement of training programme participation increases exit rates from unemployment for women looking for part-time employment and unemployed persons aged between 20 and 27 years.

Besides the mentioned studies based on randomised experiments, Jensen et al. (2003) exploit a Danish labour market reform in 1994, the Youth Unemployment Programme. Due to the reform, low-skilled people aged younger than 25 years that have been unemployed for more than 6 months during the last 9 months received an offer of a special vocational education, and received only 50 per cent of their unemployment benefit while participating in this training. If they refused to participate or to enter the ordinary education system, they were sanctioned by a loss of their unemployment benefit. The authors used survey data to estimate separately announcement, direct programme and sanction effects on the transition rate out of unemployment. They neither found announcement effects on the transition rate into schooling nor on the transition rate into employment.

Overall, evidence on ALMP announcements is still limited with little evidence on Germany and none on workfare announcements for the group of welfare recipients so far. In contrast to most existing studies, we not only investigate labour market outcomes, but also open the black box by studying changes in job search behaviour and reservation wages directly. We study the intention-to-treat effect of One-Euro-Jobs. As more than half of the welfare recipients receiving a One-Euro-Job announcement also participate in this One-Euro-Job before the next interview, this captures announcement as well as participation effects. For those who actually participate, participation reduces the time available for job search. Moreover, the short-term effect depends on the welfare recipient’s perception of the programme. How welfare recipients perceive an ambiguous programme like One-Euro-Jobs is not straightforward to predict. Considering that One-Euro-Jobs can be used as a work test, we could expect positive overall effects on the job search intensity and negative effects on the reservation wage. However, the perception could also be positive because at least some participants derive utility from participation [e.g., in terms of improved employment prospects (Hohmeyer and Wolff 2012), social integration (Gundert and Hohendanner 2015) or well-being (Knabe et al. 2017)]. Therefore, the reservation wage could also increase and job search intensity decrease. Overall, the effect of receiving a One-Euro-Job announcement is not clear a priori, but has to be estimated.

## Data and method

### Data and sample design

Our analyses are based on survey data from the first seven waves of the Panel Study ‘Labour Market and Social Security’ (PASS) [for a description see Trappmann et al. (2013)]. The PASS provides annual survey data on topics such as unemployment, poverty and the social situation of households receiving welfare benefits. We use the subsample of the PASS consisting of households that were drawn from the administrative data covering the population of welfare recipient households.

**Fig. 1:**
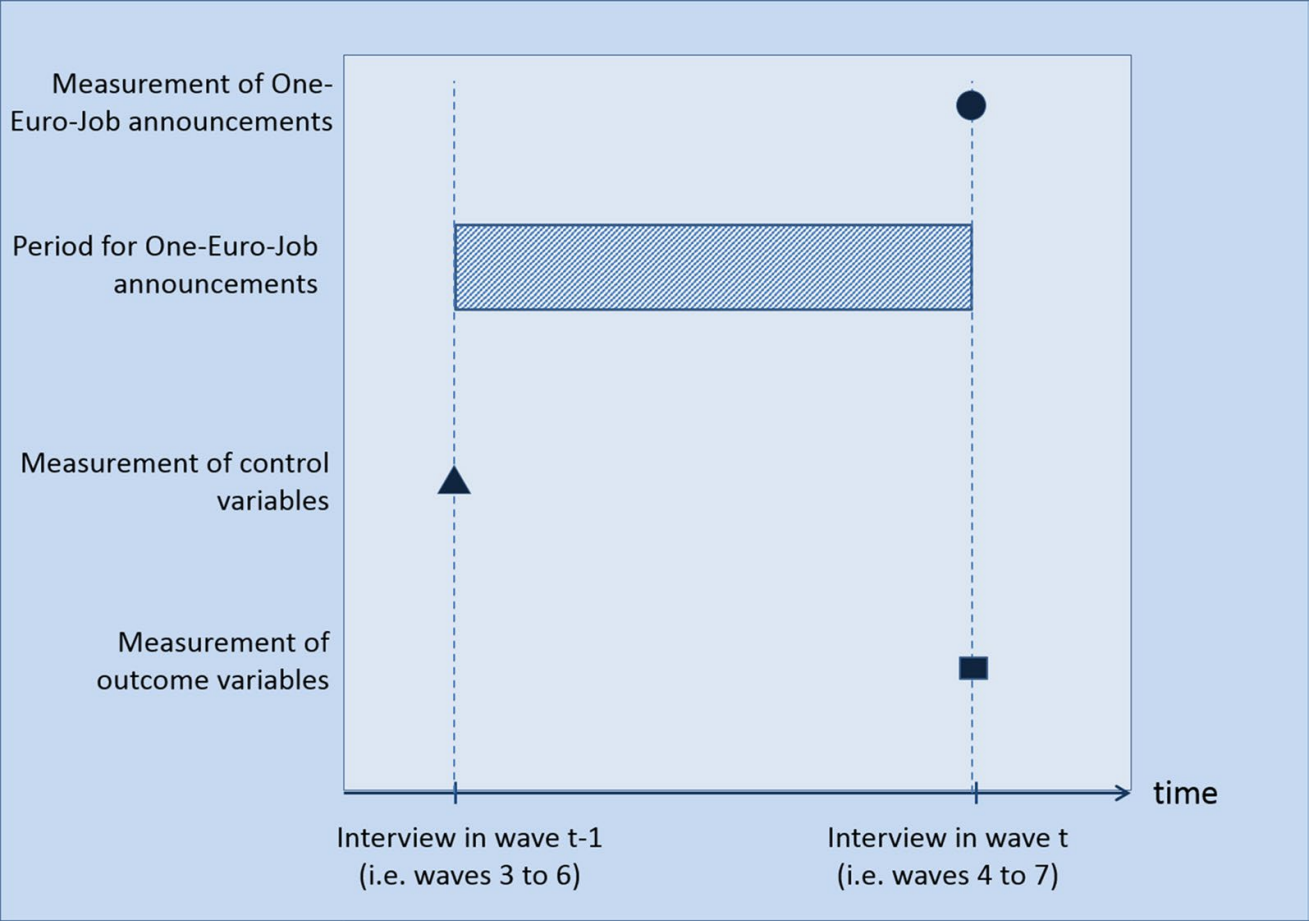
Design of the study

The PASS surveys One-Euro-Job announcements and participations from wave four onwards. Persons (excluding pupils/students) aged between 15 and 64 years who live in a household that received welfare benefits for some time during the last year are asked, whether and in which month the job centre announced a particular One-Euro-Job to them that they should participate in.^5^ Whether this announcement was in written or oral form is left open. Consequently, waves four to seven contain information on the One-Euro-Job announcements between the current and the previous wave (waves three to six) (see Fig. 1 on the design of the study).^6^ To ensure that the covariates in our models are independent of treatment, they are measured at the time of the interview of the previous wave, before the reported announcement could have taken place. Therefore, our final sample only comprises individuals who were interviewed in two subsequent waves. The outcomes variables are measured at the second of the two waves.

We selected the sample as follows: We started with 21,493 observations of respondents to waves 4–7 who were also interviewed in the previous wave (Table 2). The questions concerning One-Euro-Jobs were posed to the cases (without pupils/students), who received UB II at some point in time during the previous year, because only those qualify for a One-Euro-Job and thus could have received a One-Euro-Job announcement. This reduces the sample to 12,493 observations. In the next step, we restricted the sample to the 7831 observations who were at risk of receiving a One-Euro-Job announcement at the time of the previous interview, i.e. were receiving welfare benefit and were principally available for One-Euro-Job participation (not employed, not in education, not retired or on maternity/parental leave). After excluding observations with missing information as well as observations starting a contributory job, i.e. a job subject to social insurance contributions, or leaving benefit receipt before the (hypothetical) announcement date (see next Sect. 4.2), we are left with 5261 observations. 677 (12.9%) of these observations received a One-Euro-Job announcement since the previous interview (Table 3).^7^

**Table 2:** Sample selection. Source: PASS_0613_v1, Statistics Department of the German Federal Employment Agency

	Observations excluded	Observations remaining
Total number of observations waves 4–7		31,099
Thereof with interview in previous wave		21,493
Observations excluded due to		
Questionnaire design of One-Euro-Job module		
Student	963	
No UB II receipt since previous wave	7940	
One-Euro-Job questions not posed by mistake	97	
		12,493
Excluding individuals not at risk of One-Euro-Job announcement at the time of the previous interview due to…		
Current One-Euro-Job participation	483	
No UB II receipt	1577	
In school, education, (alternative) military service	610	
Maternity/parental leave	177	
Retired	172	
Sick, incapable of working	61	
Contributory employment	1582	
		7831
Missing information concerning…		
(Date of) One-Euro-Job announcement	43	
Covariates	574	
Outcomes variables	1086	
End of UB II receipt or start of employment before (random) announcement date	867	
Remaining number of observations		5261

**Table 3:** Number of observations by wave. Source: PASS_0613_v1, Statistics Department of the German Federal Employment Agency

	Wave 4	Wave 5	Wave 6	Wave 7	Total
Number of observations	1164	1257	1492	1348	5261
Thereof with One-Euro-Job participation	222	181	145	129	677
(in %)	19.1	14.4	9.7	9.6	12.9

### Method and operationalization

We applied propensity score matching to estimate the intention-to-treat effect of receiving a One-Euro-Job announcement since the previous interview on the job search behaviour, reservation wage and labour market performance. The basic idea of this approach is to compare the outcomes of treated individuals (people who received a One-Euro-Job announcement between two subsequent interviews), to non-treated individuals comparable in all relevant characteristics influencing both treatment status and outcomes (Roy 1951; Rubin 1974). Our parameter of interest is the average treatment effect on the treated (ATT)

$$E(Y_{i}^{1} - Y_{i}^{0} |D = 1)$$

which is the expected difference in the outcomes in case of treatment $Y_{i}^{1}$ and non-treatment $Y_{i}^{0}$ for treated (D = 1). An advantage of using this approach over methods like OLS is that we do not have to make any assumptions on the functional form of the outcome equation and do not include control individuals who on the basis of their personal characteristics are hardly comparable to the treated individuals.

The crucial, non-verifiable assumption is that we observe all relevant aspects and selection into treatment is solely on observables [conditional independence assumption (CIA)]. If the CIA holds, the ATT can be estimated by first computing for each treated person the difference between her or his outcome and the average outcome of the matched controls and then by computing the mean of these differences.

We use the propensity score as a balancing score. Hence, we first use a probit model to estimate the parameters of pre-treatment characteristics X that determine a treatment equation and predict each individual’s treatment probability. We then match treated and controls on their predicted treatment probability using algorithms of nearest neighbour matching with replacement and radius-calliper matching (Rosenbaum and Rubin 1983). We thus select for each treated person controls with the lowest (absolute) differences between their propensity score and the one of the treated person. This approach requires observations to have a participation probability larger than 0 and smaller than 1 (common support). Furthermore, the distributions of the participation probabilities of treated and potential controls have to overlap such that for each participant there is a sufficient number of non-participants with similar propensity score values.

To make sure that effects are not biased because control individuals enter employment and/or leave welfare very early after the interview, we computed hypothetical announcement months for members of the control group, randomly drawn from the distribution of announcement dates of the treatment group (for a description of the procedure see Appendix A1). Respondents who between the interview and their (hypothetical) announcement date already successfully found contributory jobs or exited benefit receipt (even temporarily) were excluded from the analyses.

The PASS allows us to control for a large variety of pre-treatment characteristics that determine treatment probability as well as labour market performance and job search behaviour outcomes. More specifically, we control for sociodemographic characteristics (e.g., age, migration status), household composition, labour market status and history of the individual and the partner and regional information (i.e. on the job centre and the labour market situation from the Statistics Department of the German Federal Employment Agency). In contrast to evaluation studies based on administrative data, we can also control for soft factors such as attitudes towards work, previous reservation wage, deprivation, life satisfaction or perceived social integration. Taken together, these variables should also very closely proxy unobserved determinants of the treatment and the outcomes, like the motivation to find work. When selecting the variables, we proceeded as follows: In the first step, we defined potentially relevant variables based on theoretical considerations, previous evidence and institutional knowledge. We decided to definitely use those variables that can be expected to influence the probability of receiving an announcement, job-search behaviour and labour market success on theoretical grounds (e.g., gender, age, migration status, education, and household context). For other variables (such as attitudes towards work, previous reservation wage, deprivation, life satisfaction, information on the partner and information on the regional labour market), we applied Wald tests and excluded the variables that were highly insignificant. To test the sensitivity of results, we performed a robustness check based on all reasonable covariates available (see Sect. 5.5). Given the large variety of information, we are confident that the CIA holds and differences in outcomes between treated and matched controls can be traced back to treatment. To provide an example of the selected covariates, Appendix A2 displays the results of the probit estimates for the main model.

Results on the announcement effects displayed in Sect. 5 are based on radius calliper matching with exact matching on gender, region and wave and with a calliper as the 99th percentile of the absolute differences between the propensity score of treated and matched controls resulting from nearest neighbour one-to-one matching with replacement (calliper for the main group is 0.014517).^8^ We study the intention-to-treat effect on different outcomes concerning job search behaviour, reservation wage, employment and income. To learn about effect heterogeneity, we estimated the effects by region and time since the end of the last contributory job.

## Results

### Selectivity of One-Euro-Job announcements

To give an impression of the selectivity of One-Euro-Job announcements, we highlight some selected results of the probit estimates of the participation equation and from the shares among controls before and after matching (Appendix A2). In line with previous evidence on the selectivity of One-Euro-Jobs (e.g., Hohmeyer and Kopf 2009), we find that women in West Germany have a lower probability of receiving a One-Euro-Job announcement than the other groups. Given the reduced inflow numbers into One-Euro-Jobs in recent years, it is not surprising that the participation probability decreases with more recent waves.

Furthermore, we find some support that One-Euro-Jobs indeed target welfare recipients with particular difficulties finding a job. First, we turn to schooling degrees: Welfare recipients without a degree/with other degree/information missing or with an intermediate degree have a higher probability of receiving an announcement than welfare recipients with a secondary schooling degree. Likewise, individuals without an occupational degree are more likely to receive a One-Euro-Job announcement than those with a vocational training degree. Second, welfare recipients whose last contributory job ended 6 or more years ago are more likely to receive an announcement than those whose last job ended less than 2 years ago.

Moreover, One-Euro-Job announcements target individuals who generally draw the attention of job centres. Announcements are more likely for welfare recipients who signed an integration agreement, who are obliged to search for a job and who were in contact with the job centre more than twenty times during the previous year.

### Matching quality

One condition for successful matching is that the distributions of the propensity scores of treated and controls overlap. Appendix A3 shows that differences in the shape of the distribution of the propensity score can be observed in some cases, but nevertheless there is sufficient mass among non-participants for regions of the propensity score with mass among participants.

Before presenting results on the intention-to-treat effects, we show that the balancing of the relevant variables between treated and matched controls succeeded. The standardized absolute bias measures the distance in the marginal distribution of the covariates. Before matching, the mean standardized absolute bias (MSB) ranges from 10 to 13 (Tables 4, 5 and 6). After Matching, the MSB is reduced to numbers below four. There is no theoretically defined threshold below which a value of the MSB implies a success of a matching procedure. However, following Caliendo and Kopeinig (2008), a reduction to values between three and five is in most studies regarded as sufficient.

**Table 4:** ATT of receiving a One-Euro-Job announcement and mean outcomes for matched controls. Source: PASS_0613_v1, Statistics Department of the German Federal Employment Agency

	Mean outcome for matched controls	ATT
Job search activities during past 4 weeks		
Job search: yes	0.544	0.047**
Job search via…		
… Job advertisements in newspapers	0.441	0.046**
… Employment agencies’ online job market	0.351	0.012
… Other internet sources	0.363	0.029
… Family and friends	0.355	0.058***
… Placement officer at the employment agency	0.282	0.020
… Private job placement service	0.107	− 0.000
… Other, not coded	0.030	− 0.001
No. of different search channels	1.987	0.192**
Looked for any possible job	0.290	0.051**
Application activities during past 4 weeks		
Replied to job advertisements	0.363	0.031
Placed an ‘employment wanted’ advertisement	0.031	− 0.001
Asked for a job at the company itself	0.313	0.049**
Submitted application without concrete job advertisement	0.235	− 0.008
Job application > 100 km away from current residence	0.061	0.001
No. of applications for job advertisements	1.916	− 0.179
No. of pro-active applications	1.043	− 0.014
Total no. of applications	2.959	− 0.193
No. of application channels	0.943	0.072
Reservation wage, employment and income		
Hourly reservation wage after tax in €^a^	6.836	− 0.228**
Contributory employment	0.071	− 0.012
Minor employment	0.187	− 0.028
Household receives UB II	0.917	0.017
Equivalent household income in €^a, b^	707.456	− 20.207
Treated	677	
Treated on support	654	
Potential controls	4584	
Matched controls	4152	
MSB before matching	9.98	
MSB after matching	1.07	

Effects on share of a positive answer (0 = no, 1 = yes) unless stated otherwise

^a^Deflated to price level 2010 = 1

^b^This income measure covers all types of income sources, also from social benefits. For details see Berg et al. (2013)

*p < 0.10; **p < 0.05; ***p < 0.01

**Table 5:** ATT of receiving a One-Euro-Job announcement and mean outcomes for matched controls by region. Source: PASS_0613_v1, Statistics Department of the German Federal Employment Agency

	East Germany		West Germany	
	Mean	ATT	Mean	ATT
Job search activities during past 4 weeks				
Job search: yes	0.567	0.035	0.530	0.036
Job search via…				
… Job advertisements in newspapers	0.435	0.050	0.439	0.042
… Employment agencies’ online job market	0.336	0.045	0.345	− 0.010
… Other internet sources	0.349	0.021	0.362	0.053*
… Family and friends	0.389	0.046	0.333	0.056*
… Placement officer at the employment agency	0.318	0.005	0.234	0.042
… Private job placement service	0.114	− 0.003	0.092	− 0.003
… Other, not coded	0.033	0.007	0.021	− 0.003
No. of different search channels	2.061	0.192	1.874	0.192
Looked for any possible job	0.348	− 0.001	0.253	0.073**
Application activities during past 4 weeks				
Replied to job advertisements	0.347	0.010	0.377	0.054*
Placed an ‘employment wanted’ advertisement	0.033	0.001	0.025	0.001
Asked for a job at the company itself	0.364	0.023	0.281	0.051*
Submitted application without concrete job advertisement	0.244	0.009	0.225	− 0.009
Job application > 100 km away from current residence	0.057	0.007	0.066	− 0.010
No. of applications for job advertisements	1.684	− 0.354	2.161	− 0.051
No. of pro-active applications	0.999	− 0.117	1.034	0.171
Total no. of applications	2.683	− 0.471	3.195	0.120
No. of application channels	0.989	0.042	0.909	0.097
Reservation wage, employment and income				
Hourly reservation wage after tax in €^a^	6.255	− 0.140	7.245	− 0.211
Contributory employment	0.076	− 0.012	0.063	− 0.010
Minor employment	0.190	− 0.008	0.172	− 0.021
Household receives UB II	0.908	0.025	0.920	0.014
Equivalent household income in €^a, b^	656.750	− 13.710	750.136	− 18.186
Treated	325		352	
Treated on support	297		337	
Potential controls	1572		3012	
Matched controls	1322		2698	
MSB before matching	10.154		10.048	
MSB after matching	2.653		1.537	

Effects on share of a positive answer (0 = no, 1 = yes) unless stated otherwise

^a^Deflated to price level 2010 = 1

^b^This income measure covers all types of income sources, also from social benefits. For details see Berg et al. (2013)

*p < 0.10; **p < 0.05; ***p < 0.01

**Table 6:** ATT of receiving a One-Euro-Job announcement and mean outcomes for matched controls by time since last employment. Source: PASS_0613_v1, Statistics Department of the German Federal Employment Agency

	Last regular job ended			
	< 4 years ago		≥ 4 years ago, never employed, missing	
	Mean	ATT	Mean	ATT
Job search activities during past 4 weeks				
Job search: yes	0.563	− 0.007	0.522	0.069**
Job search via …				
… Job advertisements in newspapers	0.451	0.022	0.427	0.063**
… Employment agencies’ online job market	0.340	0.009	0.358	− 0.002
… Other internet sources	0.379	0.012	0.351	0.036
… Family and friends	0.360	0.037	0.334	0.075***
… Placement officer at the employment agency	0.272	0.012	0.292	0.004
… Private job placement service	0.119	− 0.018	0.094	0.009
… Other, not coded	0.033	0.003	0.026	0.000
No. of different search channels	2.000	0.083	1.941	0.215*
Looked for any possible job	0.295	− 0.035	0.293	0.070***
Application activities during past 4 weeks				
Replied to job advertisements	0.408	− 0.029	0.350	0.048*
Placed an ‘employment wanted’ advertisement	0.029	− 0.012	0.038	− 0.002
Asked for a job at the company itself	0.330	− 0.005	0.290	0.077***
Submitted application without concrete job advertisement	0.260	− 0.023	0.233	− 0.013
Job application > 100 km away from current residence	0.084	− 0.007	0.055	0.000
No. of applications for job advertisements	2.303	− 0.303	1.813	− 0.196
No. of pro-active applications	1.171	0.362	0.987	− 0.165
Total no. of applications	3.474	0.059	2.800	− 0.360
No. of application channels	1.027	− 0.069	0.911	0.109*
Reservation wage, employment and income				
Hourly reservation wage after tax in €^a^	6.801	− 0.059	6.806	− 0.238*
Contributory employment	0.129	− 0.023	0.049	− 0.010
Minor employment	0.178	− 0.047	0.190	− 0.016
Household receives UB II	0.857	0.037	0.943	0.011
Equivalent household income in €^a, b^	712.449	25.188	709.798	− 37.383
Treated	192		478	
Treated on support	169		455	
Potential controls	1504		3044	
Matched controls	1134		2650	
MSB before matching	13.146		11.100	
MSB after matching	3.659		1.702	

Effects on share of a positive answer (0 = no, 1 = yes) unless stated otherwise

^a^Deflated to price level 2010 = 1

^b^This income measure covers all types of income sources, also from social benefits. For details see Berg et al. (2013)

*p < 0.10; **p < 0.05; ***p < 0.01

Furthermore, we used t-tests to check the balancing of the single covariates after matching. There are no statistically significant differences in means of covariates between treated and matched controls after matching (results are available on request).

### Main analyses

We estimated the effects of receiving a One-Euro-Job announcement on different job search and application activity outcomes, the reservation wage, income and employment. Treatment effects of our propensity score matching analyses and mean outcomes for matched controls are displayed in Table 4.

As a first group of outcomes, we consider different aspects of job search behaviour. These job search activities (and the application activities discussed below) are surveyed for the period of the 4 weeks prior to each interview. First of all, we find that a One-Euro-Job announcement increases the probability of active job search by 4.7 percentage points from a base level of 54%. More specifically, we find an increased likelihood of between four and six percentage points for job search via newspapers and family and friends, but no increased probability of using employment agency resources (via internet or placement officers) for job search due to a One-Euro-Job announcement. The number of different job search channels used increases by about one-fifth (0.19).

For application activities, we find that receiving a One-Euro-Job announcement raises the likelihood of asking for a job at the company itself significantly by five to six percentage points. Though, the likelihood of application for a job more than 100 km away from the current residence does not increase. Apparently, there is no increased willingness to make a concession concerning commuting over a longer distance (or moving). However, the likelihood of looking for any possible job increases by five percentage points from a base level of 29% for the matched controls.

Data on the hourly reservation wage show that an announcement of a One-Euro-Job decreases the hourly reservation wage by €0.23 from a base level of €6.84, which is already quite low.

Concerning the employment situation, we do not find One-Euro-Job announcements to lead to (well-determined) changes in the likelihood of being in contributory or minor employment.^9^ This result indicates that the increased job search intensity and willingness to make a concession do not lead to employment gains in the very short term for welfare recipients. Moreover, we have to consider that more than half of the welfare recipients receiving an announcement also start to participate in the programme. Therefore, lock-in effects are likely as well.

With respect to the income situation, we find that the overall equivalent income (including benefits) tends to decrease by €20 from a base level of €707, while the likelihood of receiving welfare benefits tends to increase slightly by 1.7 percentage points from a high base level of 92% (both not significant).

Overall, receiving a One-Euro-Job announcement increases several aspects of job search intensity and applications activities. Also, the willingness to make a concession with respect to the reservation wage as well as to searching for any job increases, but not with respect to looking for a job more than 100 km away from the current residence. These effects on the job search behaviour and the willingness to make a concession do not lead to employment gains in the short term.

Table 4 displays results on the significance of several effects, which were tested independently on the hypothesis of an effect of zero. The more hypotheses are taken into account, the larger is the probability that at least one test result will be significant (Sankoh et al. 1997). With respect to the ten outcomes on “Job search activities during past 4 weeks”, the probability that the effect on at least one outcome would be significant at a 5 per cent level would become 1 − (1 − 0.05)^10^ = 0.401. Hence the probability of a type one error (false rejection) would be far larger than if there was only one outcome in this family. To take this issue into account, we computed familywise adjusted p-values following Tukey et al. (1985).^10^ The original p-values of the effect estimates are reduced, which in turn reduces the just mentioned probability of a type one error. This was done separately for the three families of outcomes: “Job search activities during past 4 weeks”, “Application activities during past 4 weeks” and “Reservation wage, employment and income”. As a result of this exercise, the effects for the following outcomes remain statistically significant at least at a 10 per cent level: job search via family and friends, looked for any possible job, asked for a job at the company itself and hourly reservation wage after tax in €. Hence, even after proceeding family wise the effects point to more intense job search and a decrease of the reservation wage.

### Subgroup analyses

The comparatively small number of observations limits our opportunities of studying effect heterogeneity. Due to the small sample sizes, estimates of the treatment effects in the subgroups are more frequently not and/or less well-determined than for the entire sample. The 95% confidence intervals of the estimated treatment effects (not displayed) indicate no significant differences between the effects for different groups. Therefore, we only briefly discuss selected results of our subgroup analyses.

To capture the differences in the labour market situation between East and West Germany, we split the sample by region (Table 5). We find that One-Euro-Job announcements increase job search and application activities in West Germany, but not in East Germany. In particular, they raise the likelihood of looking for any possible job in West Germany by seven percentage points. As the initial willingness to look for any possible job is lower in West Germany [25% compared to 35% (East)], there might be more scope for adjustments due to treatment. Results of an analysis with familywise adjusted the p-values as described for the main analyses imply that for West Germany only the effect estimate on the outcome looking for any possible job remains significant.

To capture one aspect of job finding prospects, we split the sample into two groups by the duration since the end of their last contributory job (less than 4 years; equal to or more than 4 years, never employed, information missing). We find that those who have not been employed for 4 years or more or have never been employed respond to a One-Euro-Job announcement by increasing their job search and application activities (Table 6): Apparently persons who have not been employed for a very long time, become motivated to look for jobs again. Also a significant reduction of their reservation wage by €0.24 from a base level of €6.81 occurs. The analysis with familywise adjusted p-values implies that for the group equal to or more than 4 years, never employed, information missing of the original nine significant effects those on job search, job search via job advertisements in newspapers, via family and friends and looked for any possible job remain significant after the adjustment.

### Robustness of results

To check the robustness of our results, we conducted different robustness checks. First, we applied different matching algorithms, such as nearest neighbour matching with five neighbours and with one neighbour (in both cases with replacement) and radius calliper matching with different callipers. Here, we would like to mention the results from nearest neighbour 5-to-1-matching with exact matching on gender, region and wave (Appendix A4).

The effects for the main group are qualitatively robust with effects of similar sizes. Concerning the subgroups, we find that it is still welfare recipients in West Germany and those who have not been employed for 4 years or more or have never been employed who respond to a One-Euro-Job announcement by increasing their job search and application activities. Occasional changes in the level of significance occur. For West German participants, the negative effect on the household equivalent income becomes larger and significant.

Second, individuals might respond differently to a One-Euro-Job announcement if it is the second or third than if it is their first announcement. Therefore, we limited the analyses to those observations for whom we did not observe an announcement before (Appendix A5). The results are qualitatively robust with effects of similar sizes. Not surprisingly, the job search effects for the sample without previous announcement are slightly larger than for the sample which includes individuals that have received an announcement before. The positive effect on the likelihood of receiving welfare benefits become significant for the total sample, for West Germans and for people who have not been employed for 4 years or more or have never been employed. Furthermore, for the main group the negative effect of finding a contributory job becomes significant.

Third, to test the robustness of the results with respect to the selection of control variables, we repeated the analyses including all available covariates (Appendix A6). The results are qualitatively robust with effects of similar sizes or slightly larger. The robustness analyses lead to results that are in line with the conclusions that we draw from the main results.

## Conclusions

The Hartz reforms in the early 2000s changed the unemployment benefit system in Germany dramatically and implied a shift towards activation. As one means of activation, a workfare programme called One-Euro-Jobs was introduced in 2005 on a large scale. So far, only knowledge on effects of actual participation on labour market performance exists. It is most likely that not only actual participation affects individual behaviour but that the mere announcement of participation does so as well. We provide first evidence on the intention-to-treat effect of receiving a One-Euro-Job announcement on job search behaviour, reservation wage and short-term employment performance of welfare recipients. The effect comprises pure announcement as well as participation effects as by the time the outcomes are measured a part of the individuals already have started or even ended the announced participation. The intention-to-treat effect may be dominated by a threat effect or an attraction effect.

Our results show that receiving a One-Euro-Job announcement increases job search and application activities. Also, the willingness to make a concession with respect to accepting any job or a lower wage increases, but not the willingness to look for a job more than 100 km away from the current residence. These results could indicate that welfare recipients on average would like to circumvent an announced One-Euro-Job participation or finish it as early as possible. As our group of participants mainly comprises hard-to-place individuals, increased job search intensity and willingness to make concessions do not lead to employment gains in the very short term.

The increased job search activities could indicate that the intention-to-treat effect of a One-Euro-Job participation is dominated by a threat effect rather than an attraction effect. One might argue though that participation itself might on average have led to more intense job search, e.g., because some participants feel that they increased their employability. Hence, the result on job search activities alone would be insufficient to state that on average a threat effect dominates an attraction effect. But, we also find that the reservation wage was reduced, which rather points to a dominance of the threat effects. These intention-to-treat effects that encompass pure announcement and participation effects have to be taken into account in a comprehensive assessment of the programme. Furthermore, our results indicate that although treatment effects are often regarded as moderate, workfare can be a useful tool for reducing the problem of moral hazard of unemployment benefit receipt. However, potential undesirable side effects on job quality have to be considered and would be an issue for future research.

The likelihood of being assigned to a programme is considerable in the observation period. Our results would most likely differ from those presented, if less or more money would have been spent on One-Euro-Jobs or on other ALMPs. This would also be the case if further conditions would be different such as the development of the German economy and of the employment stock, conditions of the supply of child care facilities. Therefore, we cannot and do not make the claim that our results would be stable under different conditions. This is a question for future research.

## Figures and Tables

**Fig. 2:**
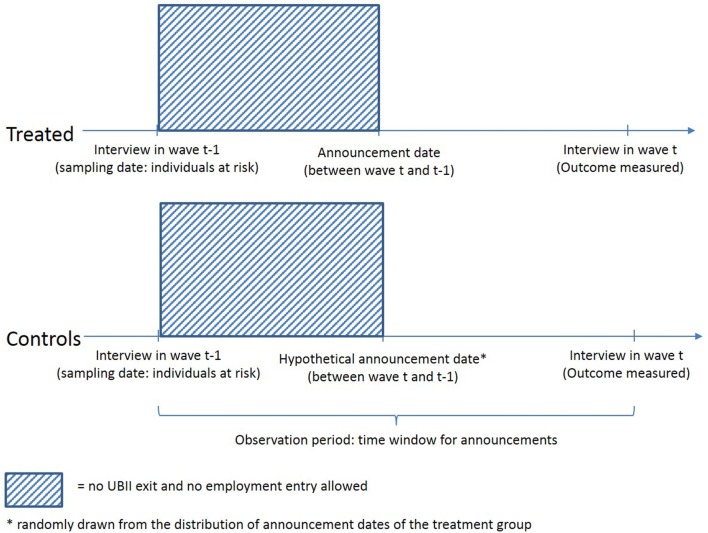
Implementation of the joining or waiting approach

**Fig. 3:**
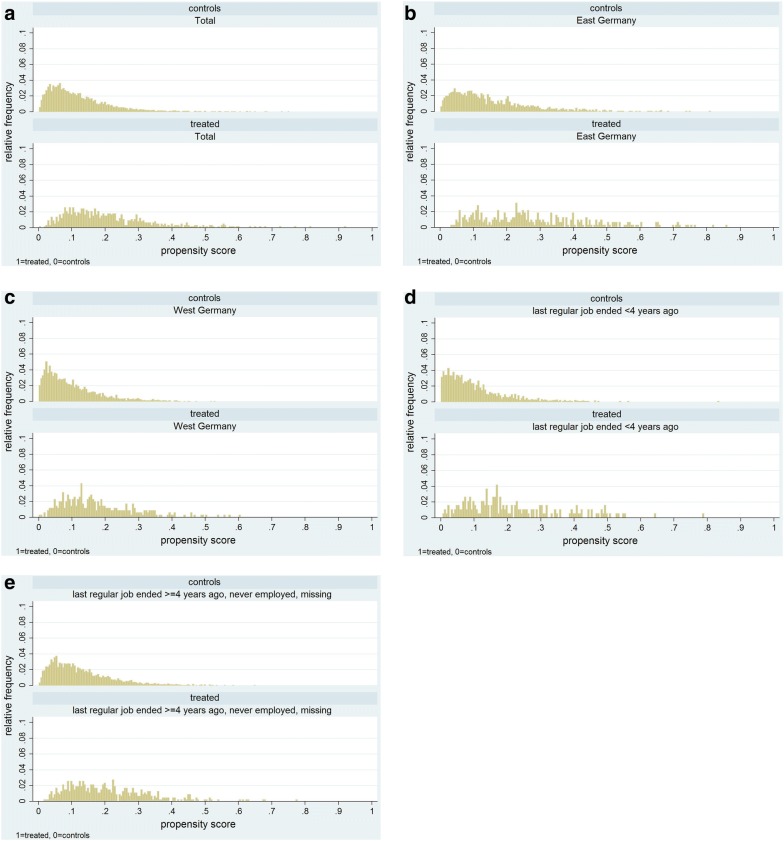
**a** Total; **b** East Germany; **c** West Germany; **d** last regular job ended < 4 years ago; **e** last regular job ended ≥ 4 years ago, never employed, missing (Source: PASS_0613_v1, Statistics Department of the German Federal Employment Agency)

## Appendix

### Appendix A1: Implementation of the random announcement date procedure

Following Sianesi (2008), we regard the treatment—receiving a One-Euro-Job announcement—as a joining or waiting decision. This approach is appropriate if eligible individuals are assigned to programmes continuously—i.e. they can be assigned now or wait and eventually be assigned later. Each eligible individual will receive treatment sooner or later provided she or he stays eligible.

The treatment group consists of individuals receiving the treatment during the chosen time interval. Non-participants are defined as waiting in the sense that they do not receive treatment until the end of the observation window, but eventually later. The estimated effect is the effect of receiving treatment versus waiting. In our case, it is the effect of receiving an announcement compared to receiving no announcement in the observation period i.e. between interviews in t − 1 and t, see also Fig. 2.

However, it could be the case that individuals do not receive an announcement because they leave welfare receipt comparatively quickly (see, e.g. Fredriksson and Johansson 2003; Lechner 1999). Therefore, we cannot just compare a treatment group who receives an announcement during the observation period to a control group receiving no announcement. This approach would imply selecting the comparison group on future (successful) outcomes. Instead, controlling for the elapsed duration of being at risk of receiving an announcement (in our case: receiving UB II and not being employed) is necessary. To make sure that effects are not biased because individuals enter employment and/or leave welfare very early after the interview, we computed hypothetical announcement months for members of the control group. These hypothetical announcement months are randomly drawn from the distribution of announcement dates of the treatment group. Respondents who between the interview and their (hypothetical) announcement date already successfully found contributory jobs or exited benefit receipt (even temporarily) were excluded from the analyses (see Fig. 2). We excluded 43 treated (6.5%) and 824 controls (15.6%) due to this: 240 started employment, 354 left welfare receipt and 273 were employed and left welfare receipt.

The estimated treated effects can be sensitive to the definition of the treatment and the control group. We would expect the effects on employment and welfare receipt to become slightly smaller and the effects on job search intensity to become slightly larger without applying a random announcement date procedure.

### Appendix A2

See Table 7.

**Table 7:** Coefficients of probit estimates and shares among controls before and after matching. Source: PASS_0613_v1, Statistics Department of the German Federal Employment Agency

	Coefficient	p-value	Mean: controls before matching	Mean: controls after matching
Female	− 0.376	0.314	0.542	0.480
West Germany	1.009	0.540	0.657	0.524
Female* West Germany	− 0.267	0.098	0.365	0.231
Wave	Reference: Wave 4 (2010)			
Wave 5 (2011)	− 0.140	0.073	0.235	0.269
Wave 6 (2012)	− 0.310	0.078	0.294	0.214
Wave 7 (2013)	− 0.264	0.094	0.266	0.191
Age in years	Reference: 15–24 years			
25–34	− 0.148	0.132	0.179	0.144
35–44	− 0.120	0.153	0.219	0.229
45–54	− 0.059	0.155	0.296	0.352
55–64	− 0.126	0.172	0.258	0.207
Migration background	Reference: none			
Information missing	0.023	0.147	0.031	0.035
Person is immigrated	− 0.026	0.096	0.174	0.131
At least one (grand-)parent immigrated	0.075	0.073	0.078	0.083
Health problems	Reference: none			
Health restrictions	0.053	0.043	0.464	0.475
Any mental disorders	0.076	0.056	0.636	0.655
School degree	Reference: secondary school			
No degree, other degree, information missing	0.355	0.093	0.077	0.113
Intermediate school	0.159	0.059	0.334	0.410
Upper secondary school	− 0.128	0.103	0.157	0.105
Occupational degree	Reference: none, semi-skilled			
Vocational training	− 0.257	0.103	0.607	0.595
University degree	− 0.036	0.175	0.089	0.076
Female*vocational training	0.162	0.199	0.324	0.296
Female*university degree	− 0.101	0.276	0.042	0.025
Time since last occupational degree (years)	Reference: up to 10 years			
Information missing	− 0.001	0.145	0.103	0.127
11–20	0.015	0.126	0.115	0.099
21–30	0.014	0.141	0.163	0.198
> 30	0.108	0.116	0.250	0.232
Female*information missing	0.522	0.248	0.052	0.065
Female*no degree	0.381	0.222	0.151	0.132
Female*11–20 years	0.412	0.220	0.062	0.049
Female*21–30 years	0.432	0.210	0.088	0.106
Female*> 30 years	0.229	0.165	0.123	0.102
Own children living in the household	Reference: none			
Child aged 0–6 years	0.002	0.136	0.157	0.120
Child aged 7–14 years	0.047	0.139	0.191	0.169
Child aged 15 or above	0.266	0.110	0.158	0.193
Female*child aged 0–6 years	0.045	0.175	0.117	0.081
Female*child aged 7–14 years	− 0.113	0.182	0.146	0.125
Female*child aged 15 years or above	− 0.142	0.128	0.116	0.136
Current status				
Providing informal care to relative or friend	− 0.014	0.086	0.091	0.087
Social engagement	− 0.029	0.065	0.269	0.257
Minor employment	− 0.349	0.067	0.234	0.152
Registered as unemployed	− 0.119	0.311	0.918	0.936
Obliged to search for a job	0.170	0.073	0.602	0.744
Frequency of job centre contact during previous year	Reference: 0–1×			
2–10×	− 0.017	0.093	0.643	0.649
11–20×	− 0.041	0.116	0.126	0.129
> 20×	0.168	0.106	0.107	0.136
Integration agreement signed	0.264	0.060	0.563	0.710
Time since last contributory job ended	Reference: < 2 years ago			
Never employed	0.052	0.121	0.074	0.076
Information missing	0.215	0.121	0.074	0.068
2–< 6 years ago	0.089	0.075	0.258	0.236
≥ 6 years ago	0.176	0.068	0.409	0.444
Occupational status in last job	Reference: blue collar			
White-collar worker	− 0.079	0.045	0.381	0.341
Else: Civil servant, self-employed, family worker	− 0.136	0.080	0.175	0.149
Unemployment during previous year	Reference: none			
Some time unemployed	0.197	0.375	0.118	0.112
Unemployed throughout the year	0.218	0.370	0.805	0.827
Some time unemployed*female	− 0.036	0.241	0.062	0.054
Unemployed throughout the year*female	− 0.093	0.217	0.425	0.388
Some time out of labour force during previous year	− 0.110	0.060	0.254	0.159
Living together with a partner	Reference: no			
Partner without further information in the data	− 0.146	0.130	0.069	0.080
Partner with further information in the data, non-married	− 0.025	0.119	0.057	0.068
Partner with further information in the data, married	− 0.498	0.110	0.201	0.138
Female*partner without further information in the data	0.134	0.206	0.038	0.041
Female*partner with further information in the data, non-married	0.120	0.179	0.030	0.029
Female*partner with further information in the data, married	0.503	0.141	0.099	0.082
Household receives UB II for	Reference: up to 5 months			
6–11 months	− 0.070	0.156	0.054	0.039
12 months and more	0.013	0.144	0.904	0.918
Deprivation index, weighted (item sum: 11.08)	Reference: ≤ 1			
1.1–2	0.053	0.078	0.317	0.324
2.1–3	− 0.061	0.072	0.251	0.242
3.1–4	− 0.044	0.086	0.106	0.108
> 4	0.191	0.110	0.056	0.093
Regional labour market situation				
Regional unemployment rate missing	1.010	0.440	0.024	0.042
Unemployment rate*East Germany	0.145	0.022	3.885	5.685
Long-term unemployment rate*East Germany	− 0.209	0.062	1.340	1.894
Vacancy-unemployment rate*East Germany	1.382	2.079	0.024	0.029
Unemployment rate*West Germany	0.038	0.038	5.077	4.142
Long-term unemployment rate*West Germany	− 0.091	0.078	1.875	1.499
Vacancy-unemployment rate*West Germany	− 0.639	0.525	0.100	0.071
Job centre information				
Inflow rate into sanctions	− 0.033	0.046	1.513	1.393
Inflow rate into One-Euro-Jobs	0.060	0.028	0.980	1.191
Female inflow rate into One-Euro-Jobs*female	− 0.021	0.029	0.418	0.501
Inflow rate into One-Euro-Jobs aged < 25 years*aged < 25 years	0.084	0.054	0.048	0.087
Regional indicator for urban–rural ratio (BIK)	Reference: < 20,000 inhabitants			
20,000–49,999 inhabitants; struct. type 1–4	− 0.101	0.129	0.090	0.092
50,000–99,999 inhabitants; struct. type 2–4	− 0.001	0.140	0.076	0.077
50,000–99,999 inhabitants; struct. type 1	0.016	0.121	0.053	0.056
100,000–499,999 inhabitants; struct. type 2–4	0.144	0.138	0.109	0.143
100,000–499,999 inhabitants; struct. type 1	− 0.037	0.104	0.212	0.182
500,000+ inhabitants; struct. type 2–4	0.077	0.140	0.058	0.054
500,000+ inhabitants; struct. type 1	0.085	0.103	0.270	0.252
Constant	− 2.167	0.616		
Pseudo-R2	0.105			
Number of observations	5261			

Dependent variable in probit estimation: receiving a One-Euro-Job announcement

### Appendix A3: Distribution of the propensity scores for treated and controls

See Fig. 3.

### Appendix A4

See Table 8.

**Table 8:** Robustness check—ATT of receiving a One-Euro-Job announcement, results from nearest neighbour 5-to-1-matching. Source: PASS_0613_v1, Statistics Department of the German Federal Employment Agency

	Total	East Germany	West Germany	Last regular job ended	
				< 4 years ago	≥ 4 years ago, never employed, missing
Job search activities during past 4 weeks					
Job search: yes	0.041*	0.040	0.042	− 0.019	0.058**
Job search via …					
… Job advertisements in newspapers	0.046*	0.054	0.040	0.024	0.057**
… Employment agencies’ online job market	0.015	0.051	− 0.006	− 0.009	− 0.009
… Other internet sources	0.028	0.028	0.048	− 0.006	0.024
… Family and friends	0.057*	0.045	0.064**	0.025	0.067**
… Placement officer at the employment agency	0.012	0.002	0.045	0.016	− 0.001
… Private job placement service	− 0.002	− 0.009	− 0.004	− 0.015	0.007
… Other, not coded	0.003	0.013	0.001	0.001	0.002
No. of different search channels	0.190*	0.210	0.199	0.039	0.176
Looked for any possible job	0.049**	− 0.009	0.062**	− 0.046	0.062**
Application activities during past 4 weeks					
Replied to job advertisements	0.036	0.016	0.055*	− 0.045	0.034
Placed an ‘employment wanted’ advertisement	0.001	− 0.009	− 0.001	− 0.020	− 0.002
Asked for a job at the company itself	0.059***	0.032	0.056*	− 0.028	0.071***
Submitted application without concrete job advertisement	− 0.008	0.020	− 0.020	− 0.040	− 0.029
Job application > 100 km away from current residence	0.008	0.009	− 0.014	− 0.011	− 0.004
No. of applications for job advertisements	− 0.175	− 0.333	0.007	− 0.625	− 0.282
No. of pro-active applications	0.076	− 0.123	0.109	0.088	− 0.300*
Total no. of applications	− 0.099	− 0.456	0.116	− 0.537	− 0.583*
No. of application channels	0.089	0.059	0.090	− 0.132	0.074
Reservation wage, employment and income					
Hourly reservation wage after tax in €^a^	− 0.257**	− 0.094	− 0.244	− 0.107	− 0.359**
Contributory employment	− 0.015	− 0.011	− 0.015	− 0.018	− 0.017
Minor employment	− 0.029	− 0.010	− 0.030	− 0.054	− 0.021
Household receives UB II	0.018	0.027	0.016	0.035	0.012
Equivalent household income in €^a, b^	− 9.858	− 10.099	− 84.859**	33.373	− 50.332
Treated	677	325	352	192	478
Treated on support	661	301	341	171	459
Potential controls	4584	1572	3012	1504	3044
Matched controls	1902	774	1079	500	1338
MSB before matching	9.976	10.154	10.048	13.146	11.100
MSB after matching	1.548	2.765	2.369	3.944	1.937

Results from nearest neighbour 5-to-1 matching with exact matching on gender, region and wave. Effects on share of a positive answer (0 = no, 1 = yes) unless stated otherwise

^a^Deflated to price level 2010 = 1

^b^This income measure covers all types of income sources, also from social benefits. For details see Berg et al. (2013)

*p < 0.10; **p < 0.05; ***p < 0.01

### Appendix A5

See Table 9.

**Table 9:** Robustness check—ATT of receiving a One-Euro-Job announcement, results for first announcements. Source: PASS_0613_v1, Statistics Department of the German Federal Employment Agency

	Total	East Germany	West Germany	Last regular job ended	
				< 4 years ago	≥ 4 years ago, never employed, missing
Job search activities during past 4 weeks					
Job search: yes	0.061**	0.044	0.035	0.019	0.069**
Job search via …					
… Job advertisements in newspapers	0.059**	0.057	0.040	0.052	0.074**
… Employment agencies’ online job market	0.015	0.030	− 0.025	0.031	− 0.014
… Other internet sources	0.034	− 0.005	0.025	0.020	0.045
… Family and friends	0.066**	0.042	0.056*	0.060	0.067**
… Placement officer at the employment agency	0.027	0.010	0.065**	0.057	0.022
… Private job placement service	0.009	− 0.009	0.015	0.025	0.014
… Other, not coded	− 0.004	0.004	− 0.008	0.001	− 0.003
No. of different search channels	0.222**	0.144	0.172	0.259	0.216*
Looked for any possible job	0.067***	0.027	0.064**	0.027	0.085***
Application activities during past 4 weeks					
Replied to job advertisements	0.034	0.055	0.045	0.007	0.046
Placed an ‘employment wanted’ advertisement	− 0.002	0.009	− 0.002	0.006	0.006
Asked for a job at the company itself	0.057**	0.030	0.071**	0.050	0.075**
Submitted application without concrete job advertisement	− 0.003	− 0.003	− 0.007	− 0.006	− 0.012
Job application > 100 km away from current residence	0.004	0.019	− 0.003	− 0.032	0.002
No. of applications for job advertisements	− 0.193	− 0.276	− 0.081	0.124	− 0.216
No. of pro-active applications	0.002	− 0.293	0.124	0.700	− 0.275**
Total no. of applications	− 0.190	− 0.568	0.043	0.824	− 0.492*
No. of application channels	0.085	0.091	0.108	0.057	0.115
Reservation wage, employment and income					
Hourly reservation wage after tax in €^a^	− 0.200	− 0.048	− 0.116	− 0.274	− 0.340**
Contributory employment	− 0.026**	− 0.016	− 0.016	− 0.053	− 0.007
Minor employment	− 0.024	− 0.054	− 0.040	− 0.045	− 0.027
Household receives UB II	0.035***	0.027	0.030*	0.077**	0.004
Equivalent household income in €^a, b^	− 16.673	− 14.543	− 21.833	− 2.571	− 26.753
Treated	530	247	283	154	369
Treated on support	502	224	272	140	347
Potential controls	4205	1403	2802	1408	2761
Matched controls	3719	1098	2224	1018	2209
MSB before matching	10.063	10.635	10.458	14.223	11.218
MSB after matching	1.626	2.834	2.281	4.999	1.695

Effects on share of a positive answer (0 = no, 1 = yes) unless stated otherwise

^a^Deflated to price level 2010 = 1

^b^This income measure covers all types of income sources, also from social benefits. For details see Berg et al. (2013)

*p < 0.10; **p < 0.05; ***p < 0.01

### Appendix A6

See Table 10.

**Table 10:** Robustness check—ATT of receiving a One-Euro-Job announcement, results from PS matching estimation with all covariates. Source: PASS_0613_v1, Statistics Department of the German Federal Employment Agency

	Total	East Germany	West Germany	Last regular job ended	
				< 4 years ago	≥ 4 years ago, never employed, missing
Job search activities during past 4 weeks					
Job search: yes	0.046**	0.022	0.046	0.046	0.078***
Job search via …					
… Job advertisements in newspapers	0.060***	0.025	0.055*	0.086*	0.079***
… Employment agencies’ online job market	0.017	0.050	− 0.002	0.003	0.010
… Other internet sources	0.029	0.029	0.060**	− 0.001	0.055**
… Family and friends	0.061***	0.040	0.057*	0.063	0.066**
… Placement officer at the employment agency	0.017	− 0.001	0.038	0.033	0.014
… Private job placement service	0.005	− 0.002	− 0.008	− 0.030	0.009
… Other, not coded	0.003	0.002	− 0.006	0.000	0.001
No. of different search channels	0.220**	0.165	0.204	0.170	0.254**
Looked for any possible job	0.054**	0.017	0.081***	0.046	0.088***
Application activities during past 4 weeks					
Replied to job advertisements	0.042*	0.006	0.067**	0.016	0.057**
Placed an ‘employment wanted’ advertisement	0.001	− 0.001	0.001	− 0.005	0.006
Asked for a job at the company itself	0.054**	0.033	0.068**	0.017	0.080***
Submitted application without concrete job advertisement	− 0.013	− 0.004	− 0.012	− 0.017	− 0.010
Job application > 100 km away from current residence	0.009	0.001	0.001	− 0.009	0.007
No. of applications for job advertisements	− 0.176	− 0.419*	0.082	− 0.127	− 0.234
No. of pro-active applications	− 0.021	− 0.191	0.153	0.470	− 0.231*
Total no. of applications	− 0.197	− 0.610	0.234	0.342	− 0.464*
No. of application channels	0.084	0.034	0.124*	0.011	0.134**
Reservation wage, employment and income					
Hourly reservation wage after tax in €^a^	− 0.303***	− 0.271	− 0.239	− 0.341	− 0.190
Contributory employment	− 0.016	− 0.006	− 0.013	− 0.021	− 0.021*
Minor employment	− 0.027	− 0.015	− 0.020	− 0.011	− 0.007
Household receives UB II	0.019	0.011	0.020	0.042	0.020
Equivalent household income in €^a, b^	− 20.960	− 15.887	− 12.672	10.298	− 49.353**
Treated	677	325	352	192	478
Treated on support	652	296	336	171	453
Potential controls	4584	1572	3012	1504	3044
Matched controls	4239	1325	2670	970	2659
MSB before matching	8.958	9.156	8.718	11.417	9.169
MSB after matching	1.383	2.727	1.760	3.745	1.413

Effects on share of a positive answer (0 = no, 1 = yes) unless stated otherwise

^a^Deflated to price level 2010 = 1

^b^This income measure covers all types of income sources, also from social benefits. For details see Berg et al. (2013)

*p < 0.10; **p < 0.05; ***p < 0.01

## Footnotes

1 Dropouts could for example be people whose job search prior to the announcement leads to a take-up of a job or vocational training, so that they do not start an announced One-Euro-Job participation.

2 The Hartz reforms aimed mainly at improving the effectiveness of public employment services and active labour market policies, activating unemployed people and raising labour demand by labour market deregulation; for details see Jacobi and Kluve (2007).

3 Own calculations for participations that started before the interview of the next wave, Source: PASS, weighted.

4 Apart from these studies, several studies are more generally concerned with ex ante effects of ALMP participation (for a review see, e.g., Andersen 2013). This includes the previously mentioned ex ante effects before an announcement of participation, which might also influence the job search behaviour of welfare recipients and which we do not measure. Despite all differences between ALMPs and benefit regimes studied, most studies find evidence that individuals change their job search and labour market behaviour when participation in an ALMP is approaching. Most studies identify a threat effect, while an attraction effect rarely is found.

5 The question is phrased as follows “Irrespective whether in such a discussion or in another situation: Was at least once [since the previous interview] a definite One-Euro-Job pointed out to you by the [job centre], in which you were supposed to participate? Please also indicate One-Euro-Jobs that you did not participate in, after all.”

6 Interviews on announcements were conducted between February 2010 and September 2013 and the reported announcements date from the period between January 2009 and June 2013. The average time interval between two interviews is just under a year (355 days). On average, One-Euro-Job announcements were surveyed 7 months after they took place.

7 Please note that individuals can be included several times in the analyses. The 5261 observations stem from 3090 individuals: 1660 are included once, 889 are included twice, 341 are included three times and 200 are included four times.

8 For comparison, we also applied nearest neighbour matching with one neighbour and with five neighbours as well as radius calliper matching using the 90th percentile of the absolute differences between the propensity score of treated and controls. Furthermore, we ran all estimations weighted by population weights. The chosen option was superior to these alternatives with respect to matching quality. Selected results of the robustness checks using nearest neighbour matching are discussed below.

9 Minor employment is a job type for which an employer and the employee are not required to pay social security contributions, and is defined by the earnings level of the employee. In our observation window, minor employment was defined in terms of earnings up to €400 a month before January 2013 and no more than €450 a month from January 2013 onwards.

10 The formula for the adjustment of the p-value is $p_{adjusted,k} = 1 - (1 - p_{k} )^{\sqrt K }$, where *p*_*k*_ is the p-value of the kth effect in a family of effects and K is the number of effects on different outcomes that belong to this family, see Sankoh et al. (1997).
